# Benthic and coral reef community field data for Heron Reef, Southern Great Barrier Reef, Australia, 2002–2018

**DOI:** 10.1038/s41597-021-00871-5

**Published:** 2021-03-16

**Authors:** Chris Roelfsema, Eva M. Kovacs, Kathryn Markey, Julie Vercelloni, Alberto Rodriguez-Ramirez, Sebastian Lopez-Marcano, Manuel Gonzalez-Rivero, Ove Hoegh-Guldberg, Stuart R. Phinn

**Affiliations:** 1 Remote Sensing Research Centre, School of Earth and Environmental Sciences, University of Queensland, St Lucia, QLD 4072 Australia; 2 Global Change Institute, School of Biological Sciences, The University of Queensland, St Lucia, QLD 4072 Australia; 3 ARC Centre of Excellence for Coral Reef Studies, School of Biological Sciences, The University of Queensland, St Lucia, QLD 4072 Australia; 4 ARC Centre of Excellence for Mathematical and Statistical Frontiers and School of Mathematical Sciences, Queensland University of Technology, Brisbane, QLD 4000 Australia

**Keywords:** Environmental impact, Biodiversity, Marine biology

## Abstract

This paper describes benthic coral reef community composition point-based field data sets derived from georeferenced photoquadrats using machine learning. Annually over a 17 year period (2002–2018), data were collected using downward-looking photoquadrats that capture an approximately 1 m^2^ footprint along 100 m–1500 m transect surveys distributed along the reef slope and across the reef flat of Heron Reef (28 km^2^), Southern Great Barrier Reef, Australia. Benthic community composition for the photoquadrats was automatically interpreted through deep learning, following initial manual calibration of the algorithm. The resulting data sets support understanding of coral reef biology, ecology, mapping and dynamics. Similar methods to derive the benthic data have been published for seagrass habitats, however here we have adapted the methods for application to coral reef habitats, with the integration of automatic photoquadrat analysis. The approach presented is globally applicable for various submerged and benthic community ecological applications, and provides the basis for further studies at this site, regional to global comparative studies, and for the design of similar monitoring programs elsewhere.

## Abstract (summary)

**Table:** 

Measurement(s)	marine benthic feature
Technology Type(s)	photoquadrat transect surveys
Factor Type(s)	benthic composition
Sample Characteristic - Organism	benthic communities
Sample Characteristic - Environment	coral reef • marine coral reef flat zone • marine coral reef crest • marine coral reef back reef • marine coral reef fore reef
Sample Characteristic - Location	Heron Island Reef, 23–052

Machine-accessible metadata file describing the reported data: 10.6084/m9.figshare.14034320

## Background & Summary

This study describes a unique point-based data set for coral reef environments, collected using a photoquadrat survey method published for seagrass environments^1^. The data set describes the spatial and temporal distribution of benthic community abundance and composition for Heron Reef, a 28 km^2^ shallow platform reef located in the Capricorn Bunker Group, Southern Great Barrier Reef (GBR), Australia. On average, 3,600 coral reef data points were collected annually over the period 2002 to 2018. Annual data sets were acquired for independent research projects, but the collection methods were consistent. The initial field data collection design was planned to acquire detailed field data to describe the spatial distribution and variability of benthic composition across the study site to assist with calibration and validation of earth observation-based mapping products.

To create a map based on earth observation imagery, it is common to use training or calibration data to transform the imagery into a map of surface properties using a supervised algorithm (e.g. multivariate statistical clustering, random forest)^2^. To report on the accuracy measures of the maps, reference or validation data are contrasted with the output maps^3^. Hence for calibration and validation purposes, georeferenced field data must be representative of all the features to be mapped and collection should ideally coincide with satellite image acquisition. Many earth observation approaches have been implemented for mapping the benthic communities of Heron Reef^4–12^ and several of these maps are now accessible online^6,13,14^.

Several studies have utilised time series benthic data to analyse changes in benthic community and coral type trends, supporting broad ecological knowledge of coral reef ecosystems such as the Caribbean reef degradation^15^ and coral cover decline on the GBR^16^. Similarly, benthic community and coral cover data sets have been identified as important indicators of coral reef health providing the backbone for monitoring and management initiatives around the world^17,18^.

Articles and data sets have been published that describe the benthic community properties of Heron Reef, however, their spatial coverage, number of georeferenced data points, and revisit times are limited^19^. The time series photoquadrat data sets presented in this paper could be used for further understanding of benthic community distribution, including statistical analysis of trends in coral cover, analysis of changes in benthic community and coral type, or used for testing of other earth observation-based mapping and modelling approaches. Additionally, as our methodology describes machine annotation of the field photoquadrats, it would be possible to reanalyse the photoquadrats with new categories not previously considered important from a biological perspective (e.g. unknown disease or impact, or a specific benthic community type), or for other features (e.g. the counting of sea cucumbers (*Holothuroidea sp.)*).

Detailed analyses of our complete data set may permit a greater understanding of the persistence and/or dynamics of the benthic community at Heron Reef. As such, our ongoing analyses include evaluation of changes in community composition following major impacts such as cyclones, coral bleaching, crown of thorns predation, etc., and additionally, statistical analyses of coral recovery after such impacts. To this degree, these benthic community data sets are invaluable.

## Methods

**Fig. 1:**
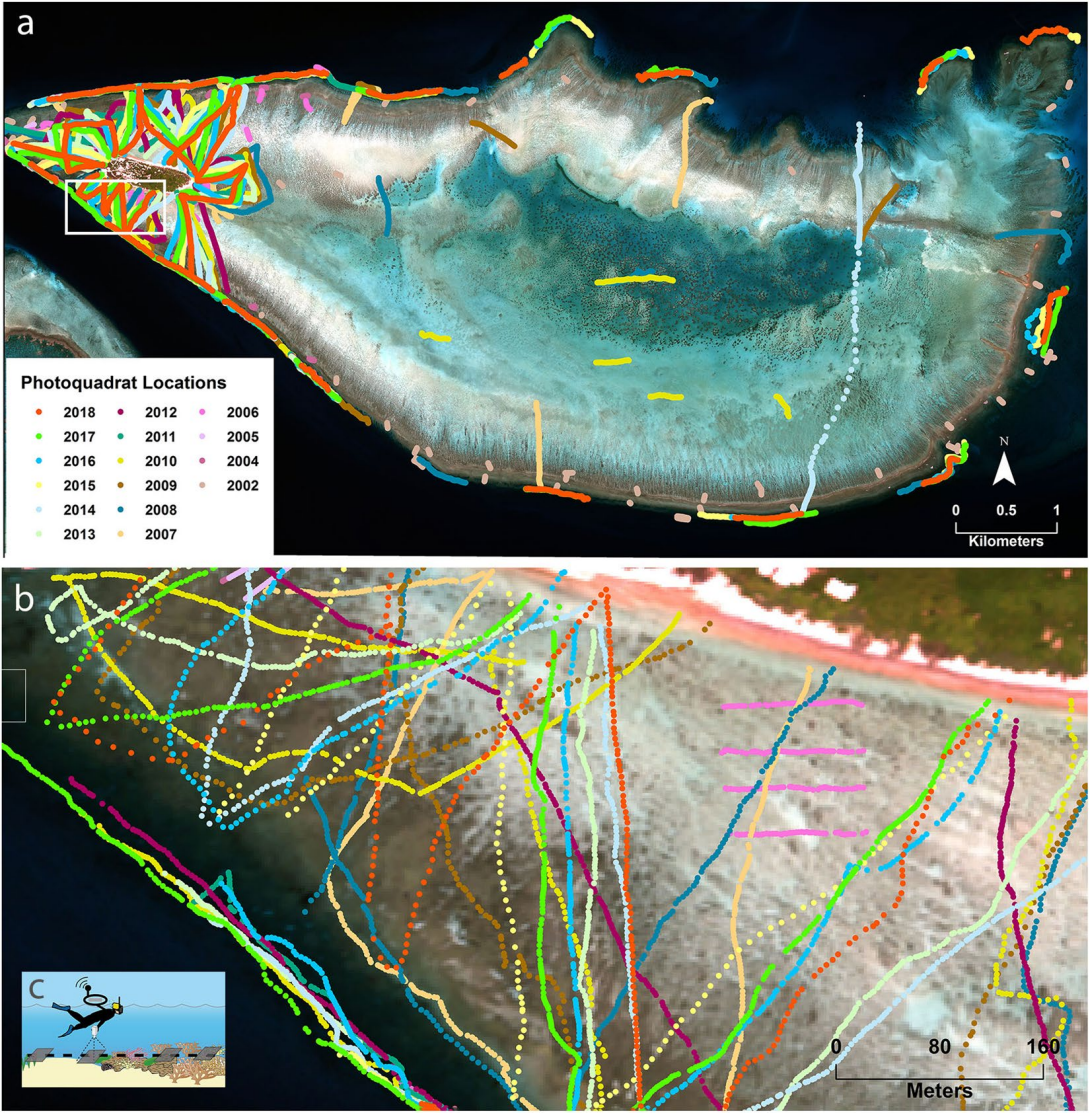
Heron Reef, southern Great Barrier Reef, Australia. (**a**) Location of photoquadrat transect surveys on Heron Reef collected over a period of 17 years, (**b**) example of the individual photoquadrat locations along the transect survey where each individual point represents a photoquadrat, and (**c**) conceptualisation of snorkeler-based georeferenced photoquadrat transect surveys.

The photoquadrat-based data in this study was collected for Heron Reef, Southern Great Barrier Reef, Australia (Fig. 1). Here we provide a short overview of the collection methods, however a detailed description can be found in^11^. These methods are applicable to any habitat. Photoquadrats were analysed for substrate and/or benthic community types known to be present on the reef (Fig. 1). The benthic community classes included in the analysis are shown in Table 1.

**Table 1:** Benthic community and coral type descriptions and their class codes used for photoquadrat annotation.

Class Code	Description	Group	Simplified Group
ACR_BRA	Acropora formosa, branching Montipera	Branching	Hard Coral
ACR_BRA_B_	Acropora formosa, branching Montipera - Bleached	Branching	Hard Coral
ACR_HIP	Acroporidae Hispidoes; thick branches, predominantly hispidose	Branching	Hard Coral
ACR_HIP_B_	Acroporidae Hispidoes; thick branches, predominantly hispidose - Bleached	Branching	Hard Coral
ACR_OTH	Acroporids with indeterminate shape, predominantly cuneiform	Branching	Hard Coral
ACR_OTH_B_	Acroporids with indeterminate shape, predominantly cuneiform - Bleached	Branching	Hard Coral
ACR_PE	Encrusting Monipora	Plate	Hard Coral
ACR_PE_B_	Encrusting Monipora - Bleached	Plate	Hard Coral
BRA_TAB_Ac	Acropora tabular/corymbose/plate	Plate	Hard Coral
BRA_TAB_B_	Acropora tabular/corymbose/plate - Bleached	Plate	Hard Coral
BRA_DIG_Ac	Acropora digitate, branches resembling fingers	Branching	Hard Coral
BRA_DIG_B_	Acropora digitate, branches resembling fingers - Bleached	Branching	Hard Coral
FAV_MUS	Favia, Favites, Platygyra, Goniastrea, Diploastrea, Lobophyllia	Massive	Hard Coral
FAV_MUS_B_	Favia, Favites, Platygyra, Goniastrea, Diploastrea, Lobophyllia - Bleached	Massive	Hard Coral
MASE_OTH	Massive, submassive, encrusting colonies of undetermined taxonomic group	Massive	Hard Coral
MASEoth_B_	Massive, submassive, encrusting colonies of undetermined taxonomic group - Bleached	Massive	Hard Coral
TFP_RDG_Al	Thin, foliose or plating colonies with visible relief structures on the plates	Plate	Hard Coral
TFP_RDG_B_	Thin, foliose or plating colonies with visible relief structures on the plates -Bleached	Plate	Hard Coral
TFP_RND_Al	Thin, foliose or plating colonies with visible rounded corallites on the plates	Plate	Hard Coral
TFP_RND_B_	Thin, foliose or plating colonies with visible rounded corallites on the plates - Bleached	Plate	Hard Coral
BRA_OTH	Branching other	Branching	Hard Coral
BRA_OTH_B_	Branching other - Bleached	Branching	Hard Coral
OTH_HC	Other HC not assigned to any other category	HC Other	Hard Coral
OTH_HC_B_	Other HC not assigned to any other category - Bleached	HC Other	Hard Coral
POCI	Pocilloporidae sp. (includes Seriatopora and Stylophora)	Branching	Hard Coral
POCI_B_	Pocilloporidae sp. (includes Seriatopora and Stylophora) - Bleached	Branching	Hard Coral
POR_BRA	Porites cylindrica, Goniopora (Poritidae branching)	Branching	Hard Coral
POR_BRA_B_	Porites cylindrica, Goniopora (Poritidae branching) - Bleached	Branching	Hard Coral
POR_ENC	Porites lichen (Poritidae encrusting)	Massive	Hard Coral
POR_ENC_B_	Porites lichen (Poritidae encrusting) - Bleached	Massive	Hard Coral
POR_MASS	Porites lobata, P. lutea (Poritidae massive)	Massive	Hard Coral
POR_MASS_B_	Porites lobata, P. lutea (Poritidae massive) - Bleached	Massive	Hard Coral
GORG	Sea Fans/Plumes; Gorgonia, Pseudopterogorgia	Soft	Other
GORG_B_	Sea Fans/Plumes; Gorgonia, Pseudopterogorgia - Bleached	Soft	Other
ALC_SF	Common large fleshy Alcyoniidae representatives	Soft	Other
ALC_SF_B_	Common large fleshy Alcyoniidae representatives - Bleached	Soft	Other
OTH_SF	Other soft coral (not sea fans)	Soft	Other
OTH_SF_B_	Other soft coral (not sea fans) - Bleached	Soft	Other
Other	All other	All other	Other
MINV_COTS	Crown of thorns sea star, Acanthaster planci	Invertebrates	Other
MOB_INV	Mobile invertebrates 1 (sea cucumber, urchin)	Invertebrates	Other
OTH_SINV	Other sessile invertebrates (zoanthids, anemones, corallimorphs, sponges, clams, etc)	Invertebrates	Other
Lobph	Lobophora; fleshy algae	Algae	Algae
Turbin	Turbinaria sp.	Algae	Algae
MAECBS	Erect Course Branching Brown: Sargassum sp.	Algae	Algae
Pad	Padina sp. (pencil shavings)	Algae	Algae
Dicsp	Dictyota sp.	Algae	Algae
Chlor	Chlorodesmis sp (turtle weed); green filamentous	Algae	Algae
MACR_Cal_H	Calicifying algae: Halimeda	Algae	Algae
Caul	Caulerpa sp., green algae	Algae	Algae
Cya_spe	Cyanobacterium sp.	Algae	Algae
ALG_OTH	Other algae	Algae	Algae
CAL_CCA_DC	Crustose Coralline Algae on dead coral	Rock	Rock
CAL_CCA_RB	Crustose Coralline Algae on rubble	Rubble	Rubble
EAM_DHC	Epithelial algal matrix smothering dead hard coral (Turf on Rock)	Rock	Rock
EAM_RB	Epithelial algal matrix smothering rubble (Turf on Rubble)	Rubble	Rubble
Sand	Sand	Sand	Sand
BMA_sand	Benthic microalgae on sand	Sand	Sand
Seagrass	Seagrass, any type	Other	Other
TAPE	Line or hardware	Other	Other
Unk	Unknown, but represents something (annotator doesn’t know what it is)	Other	Other
Unc	Unclear; point falls in a shadowy, blurry, dark area	Other	Other
WATE	Blue background	Other	Other

Manual and automated (machine) annotation utilized the full labelset (63 class codes). Following machine annotation, these 63 class codes were aggregated via broad groups into six simplified groups for validation of the machine learning.

### Georeferenced photoquadrat data collection

Detailed information on benthic community composition was gathered at Heron Reef on the reef flat (0–2 m depth) and at the 5 m contour on the reef slope using a repeatable and fine spatial scale (sampling every 2–4 m) technique for surveying benthic cover^11^. The technique required a snorkeler or diver manually capture georeferenced photoquadrats along defined transect surveys using a standard digital camera in a waterproof housing (e.g. Sony Cyber shot, Canon AA540, Lumix, or Olympus T4). A plumb-line attached to the camera, ensured that the footprint of each photoquadrat approximated 1 m^2^ of the benthos.

From 2002–2004, a 100 m transect tape was deployed at each defined survey start site at a maximum depth of 3 m, or on scuba at 5 m depth. From 2005 onwards, instead of deploying a tape, the surveyor towed a standard handheld GPS (e.g. Garmin eTrex, Garmin 72) at the surface in a waterproof bag for all surveys. This enabled accurate registration of the location of the acquisition of each photoquadrat, which was subsequently assigned via time synchronization, with the track log from the towed GPS. Once this method was established transect survey lengths were extended to distances of 500 m–1500 m. The start and end point of each transect was defined by GPS waypoints, permitting accurate revisits in subsequent years. The distance between successive photoquadrats was estimated by the surveyor’s kick cycle. However this was not considered a problem as the exact location of each photograph was known through the GPS synchronisation.

All surveys were performed during the day, and derivation of sunlight and sun angle can be ascertained through the timestamp of each photoquadrat and its corresponding GPS location. Reef Flat surveys were collected at high tide to provide sufficient water depth for the snorkeler to safely traverse the reef. Reef Slope surveys were collected at low tide. No water quality information was recorded.

The locations of the transect surveys were chosen to ensure they traversed gradients or edge features to detect any change in benthic cover over these features. This was done initially through visual assessment of existing satellite imagery in combination with expert knowledge of the study area. The aim was to produce data that provided an adequate representation of the variation in benthic community cover across Heron Reef. Limited transect surveys were located within the deep lagoonal area of the reef, as this area is hard to access by boat due to tidal range restrictions permitting short working times in the lagoon. Transect surveys were revisited in subsequent years, and additional transect surveys were included on subsequent trips based on increased knowledge of the environment. The benthic data sets and photoquadrat images are available at^20^.

### Automated photoquadrat analysis for benthic community composition

Percentage cover of the benthic communities for each photoquadrat was determined through a machine-learning (ML) approach which assessed benthic community composition. A previously devised category scheme consisting of 63 class codes that differentiated all major GBR-specific coral morphologies and other bottom types was used^21^ which, following machine annotation, were collapsed first into broad groups and subsequently into six simplified groups for validation purposes (Table 1).

Initial training of the ML platform was achieved via manual annotation of approximately 5% of the total number of photoquadrats (equivalent to 108,700 annotated points; based on^21^), to achieve a machine annotation accuracy of >70% as determined by the classifier^21^. A unique source was created for each camera used. To give a default and uniform image annotation area, boundaries of 5% were used for the top and left sides of the photoquadrat, whilst a boundary of 95% was used for the right and bottom sides of the photoquadrat. Annotation points (50) were generated randomly over the entire annotation area per photoquadrat. For manual annotation of photoquadrat sets, the level of confidence was set to 100%. A further approximately 2.5% of photoquadrats were manually annotated in an identical manner to provide a validation data set to calculate the accuracy of the machine annotation. Automated annotation of the remaining 92.5% of the photoquadrats was achieved subsequently^22^.

## Data Records

Detailed information regarding the output benthic cover percentages and the number of benthic photoquadrats acquired for each field campaign are documented in Table 2. The benthic data sets and photoquadrat images are available at^20^, with the photoquadrats and benthic cover analysis for individual survey years accessible online through the campaign specific DOIs listed in the table, from where the data can be downloaded directly.

**Table 2:** Overview of the data files that represent the 58,941 georeferenced photoquadrats captured during the field campaigns, in addition to links to the percentage benthic cover data sets generated via machine learning for each year.

Year-Month	Photoquadrats	Length of survey (m)	Benthic DOI (pangaea.de)	Photoquadrat DOI (pangaea.de)
2002–11	1965	100	10.1594/PANGAEA.907025	10.1594/PANGAEA.895556
2004–03; 2004–05	1588	100	10.1594/PANGAEA.903850	10.1594/PANGAEA.895557
2005–05	1004	100	10.1594/PANGAEA.903851	10.1594/PANGAEA.894796
2006–06	1941	300–1500	10.1594/PANGAEA.903847	10.1594/PANGAEA.895558
2007–09	2923	300–1500	10.1594/PANGAEA.903779	10.1594/PANGAEA.895563
2008–10	3608	300–1500	10.1594/PANGAEA.903788	10.1594/PANGAEA.895569
2009–11	4400	300–1500	10.1594/PANGAEA.90378	10.1594/PANGAEA.895570
2010–11	4701	300–1500	10.1594/PANGAEA.903784	10.1594/PANGAEA.894797
2011–11	3602	300–1500	10.1594/PANGAEA.904704	10.1594/PANGAEA.895157
2012–07	3903	300–1500	10.1594/PANGAEA.904706	10.1594/PANGAEA.895121
2013–11	3589	300–1500	10.1594/PANGAEA.904710	10.1594/PANGAEA.895160
2014–11	4194	300–1500	10.1594/PANGAEA.904715	10.1594/PANGAEA.895124
2015–11	4277	300–1500	10.1594/PANGAEA.904716	10.1594/PANGAEA.895147
2016–09	4197	300–1500	10.1594/PANGAEA.907013	10.1594/PANGAEA.894800
2017–11	6499	300–1500	10.1594/PANGAEA.903766	10.1594/PANGAEA.895154
2018–11	5545	300–1500	10.1594/PANGAEA.903767	10.1594/PANGAEA.899670

The complete data set is available at^20^.

## Technical Validation

To understand the validation technique applied to these data sets, it is important to reiterate the purpose of collecting the data set itself, which was a fast field method to gather benthic community information over a large spatial extent, whilst accurately representing variability. Validation of the data set was conducted on various levels, and included: standardisation of photoquadrat capture method and conditions, and a quantitative accuracy assessment.

### Standardisation of photoquadrat image capture

To standardise photoquadrat image capture, the camera and lens setup used was calibrated prior to annual survey, so as to capture a footprint that covered the same extent of the benthos. This was accomplished by attaching a plumb-line to the camera system such that when it touched the bottom, the captured photoquadrats represented ~1 m^2^ of the benthos. To do this standardisation, the camera was moved vertically over a marked 1 m^2^ until the field of view enveloped the area, and the plumb-line was fixed. During the survey the operator used the plumb-line to determine the camera height above the ground. When held vertically with the weight touching the substrate this permitted reproducible capture of photoquadrats that covered the same area for all surveys. Light conditions were generally the same for each expedition, the data collected over a consecutive 4–5 day period, with stable weather, water clarity conditions and tidal range. Ideally light conditions would have been standardised using a strobe, however this would slow down the speed of the transect surveys.

### Quantitative accuracy assessment

To determine the accuracy of the machine annotation we constructed a confusion matrix that compared, for a select set of validation photoquadrats, the benthic composition output from the machine learning annotation (modelled data), with the equivalent manual annotations (reference data). Using the confusion matrix we calculated the overall accuracy and the individual benthic label user and producer accuracy following a well-documented method^3^. All cameras demonstrated an overall accuracy of between 74% and 82% (Table 3;^3^). To provide a validation data set, ~2.5% of photoquadrats were manually annotated in an identical manner to the training data (36,950 annotated points; see Methods Section).

**Table 3:** Quantitative assessment of the machine annotation stevia construction of a confusion matrix.

Camera		SONY	Canon	Lumix	Olympus
Years		2002–2006	2007–2010	2011–2016	2017–2018
Overall Accuracy (%)		79.1	81.8	73.9	79.8
User’s Accuracy (%)	Hard Coral	79.9	83.6	83.2	88.2
	Rock	77.2	79.3	71.2	74.4
	Rubble	68.0	68.8	61.5	25.0
	Sand	85.7	90.3	87.2	93.9
	Algae	85.7	79.4	74.4	71.4
	Other	52.4	33.3	57.3	61.7
Producer’s Accuracy (%)	Hard Coral	76.0	72.7	72.5	70.2
	Rock	89.2	92.6	90.5	94.8
	Rubble	5.3	15.6	4.7	10.2
	Sand	92.1	94.5	89.8	91.8
	Algae	6.8	42.8	19.4	24.2
	Other	23.7	18.7	24.0	33.5
# Points		8,000	7,150	18,500	3,300

For each camera used, machine annotation (modelled data) of 2.5% of all the photoquadrats captured was compared with manual annotation (reference data) of the same validation data set in a using standard confusion matrix^3^. From this, the overall accuracy and individual class accuracies were calculated following a well-documented approach^3^.
